# Multilevel regulation of anthocyanin-promoting R2R3-MYB transcription factors in plants

**DOI:** 10.3389/fpls.2022.1008829

**Published:** 2022-09-06

**Authors:** Jianfei Yang, Yunzhu Chen, Zhihong Xiao, Hailong Shen, Yuhua Li, Yu Wang

**Affiliations:** ^1^State Key Laboratory of Tree Genetics and Breeding, Northeast Forestry University, Harbin, China; ^2^School of Forestry, Northeast Forestry University, Harbin, China; ^3^State Key Laboratory of Utilization of Woody Oil Resource, Hunan Academy of Forestry, Changsha, China; ^4^College of Life Sciences, Northeast Forestry University, Harbin, China

**Keywords:** anthocyanin biosynthesis, R2R3-MYB transcriptional activator, environment cues, posttranslational regulation, transcriptional regulation, integrated regulatory network, MBW complex

## Abstract

Anthocyanins are common secondary metabolites in plants that confer red, blue, and purple colorations in plants and are highly desired by consumers for their visual appearance and nutritional quality. In the last two decades, the anthocyanin biosynthetic pathway and transcriptional regulation of anthocyanin biosynthetic genes (ABGs) have been well characterized in many plants. From numerous studies on model plants and horticultural crops, many signaling regulators have been found to control anthocyanin accumulation *via* regulation of anthocyanin-promoting R2R3-MYB transcription factors (so-called R2R3-MYB activators). The regulatory mechanism of R2R3-MYB activators is mediated by multiple environmental factors (e.g., light, temperature) and internal signals (e.g., sugar, ethylene, and JA) in complicated interactions at multiple levels. Here, we summarize the transcriptional control of R2R3-MYB activators as a result of natural variations in the promoter of their encoding genes, upstream transcription factors and epigenetics, and posttranslational modifications of R2R3-MYB that determine color variations of horticultural plants. In addition, we focus on progress in elucidating the integrated regulatory network of anthocyanin biosynthesis mediated by R2R3-MYB activators in response to multiple signals. We also highlight a few gene cascade modules involved in the regulation of anthocyanin-related R2R3-MYB to provide insights into anthocyanin production in horticultural plants.

## Introduction

Anthocyanins are the red, blue and purple pigments in vegetative and reproductive tissues of plants and also act as antioxidants to scavenge ROS (reactive oxygen species) and protect plants against multiple abiotic and biotic stresses (Butelli et al., 2012; Li P. et al., 2017; Xu et al., 2017). Consumers also desire foods with anthocyanins because they provide many health benefits (Allan and Espley, 2018).

Among the structural genes involved in the pathway for anthocyanin biosynthesis, which extends from an early step in the flavonoid biosynthetic pathway that branches off the phenylpropanoid pathway, several have been well-characterized: CHALCONE SYNTHASE (*CHS*), CHALCONE ISOMERASE (*CHI*), FLAVANONE 3-HYDROXYLASE (*F3H*), DIHYDROFLAVONOL 4-REDUCTASE (*DFR*), ANTHOCYANIDIN SYNTHASE (*ANS*), and UDP-GLUCOSE:FLAVONOID 3-O-GLUCOSYLTRANSFERASE (*UFGT*; Gonzalez et al., 2008). The pathway for anthocyanin production is highly conserved, and the structural genes can be divided into two groups. The so-called early biosynthetic genes (EBGs; i.e., flavonoid biosynthetic genes such as *CHS*, *CHI* and *F3H*), which encode the enzymes involved in the production of the common colorless precursors, and the late biosynthetic genes (e.g., *DFR*, *ANS*, *UFGT*), which encode the enzymes that catalyze reactions to produce the various anthocyanins. The expression of anthocyanin biosynthetic genes (ABGs) is mainly upregulated by the transcription complex MYB-bHLH-WD40 (the MBW complex). Variations in the spatial and temporal expression of R2R3-MYBs and bHLHs that comprise the complex are responsible for the various patterns of anthocyanin accumulation during plant development (Gonzalez et al., 2008; Lloyd et al., 2017; Wang et al., 2019).

The main MYB transcriptional activators involved in the MBW complex belong to subgroup 6 (SG6) of R2R3-MYBs, e.g., MYB75/PRODUCTION OF ANTHOCYANIN PIGMENT1 (PAP1), MYB90/PAP2, MYB113 and MYB114 in *Arabidopsis* (Gonzalez et al., 2008), MdMYB10/1 in apple (Espley et al., 2009), SlAN2-LIKE in tomato (Sun et al., 2020), PyMYB10 in pear (Qian et al., 2014), DcMYB7 in carrot (Xu et al., 2019) and Ruby1 in orange (Butelli et al., 2012). The mechanism by which MBW complex regulates anthocyanin biosynthesis, especially the roles of MYB transcription factors (both MYB activators and repressors), have been discussed in many excellent review articles (Xu et al., 2015; Chaves-Silva et al., 2018; Naing and Kim, 2018). In brief, the R2R3-MYB activators bind to flavonoid-related MYB-binding elements in the promoter of the ABGs and activate their expression by forming an active MBW complex to promote anthocyanin biosynthesis. Although it is known that expression of R2R3-MYB activators is regulated by multiple signals, the upstream regulators and signaling cascades involved in the signaling transduction pathways that control the activity of R2R3-MYB activators are poorly understand so far.

Recently, the transcriptional and posttranslational regulatory mechanisms of R2R3-MYB activators in response to multiple environmental stimulus have been elucidated. In addition, a few components have been identified as integrators of environmental and internal signals to regulate anthocyanin accumulation in *Arabidopsis* and in various horticultural plants. In the present review, we provide an overview of the multilevel regulation of these R2R3-MYB activators and focus our discussion on several signaling cascades responsible for the integration of environmental cues and hormone signaling pathways that regulate anthocyanin accumulation.

## Regulation of R2R3-MYB activators at the transcriptional level

The expression level of the many R2R3-MYB transcriptional activators that have been identified in plants is highly correlated with the anthocyanin content in the organ or tissue (Espley et al., 2009; Castillejo et al., 2020; Sun et al., 2020). Though the transcriptional controls of R2R3-MYB activators are complicated, the regulatory model includes three aspects: upstream regulation of the R2R3-MYB activators by transcription factors (TFs), natural variations in the promoter, and epigenetic regulation.

### Regulation of R2R3-MYB activators by upstream transcription factors

The transcriptional regulation of anthocyanin-related R2R3-MYB activators by upstream TFs is controlled by hierarchical regulatory networks in response to multiple stimuli. Several bZIP, WRKY, NAC, ERF/AP2, HD-ZIP TFs involved in various signaling pathways have been identified as upstream regulators of the anthocyanin-related R2R3-MYBs (Table 1). Some of these TFs control the transcript levels of R2R3-MYB activators by directly binding to the *cis*-elements in the promoter region of the gene, while the other TFs regulate expression of R2R3-MYB activators indirectly, probably by interacting with other upstream TFs or by modulating their expression levels.

**Table 1:** Regulators involved in anthocyanin biosynthesis by targeting R2R3-MYB activators in plants.

Factors	Descriptions	Mode of regulation	Species	Target MYB genes	References
Transcription regulators					
HY5	Bind to G-box or ACE-box	Positive	*Arabidopsis*	AtPAP1, AtPAP2	Wang et al. (2020)
			Apple	MdMYB10	An et al. (2017)
			Tomato	SlAN2-like, SlAN2	Qiu et al. (2019)
			Pear	PyMYB10	Wang et al. (2020)
			Eggplant	SmMYB35	Li L. et al. (2021)
BBX20/21/22	Form active HY5-BBX complex	Positive	*Arabidopsis*	AtPAP1, AtPAP2	Xu et al. (2016)
			Apple	MdMYB1/10	An et al. (2019) and Fang et al. (2019b)
			Pear	PpMYB10	Bai et al. (2019)
			Rice	OsC1	Bai et al. (2019)
MdbZIP4-like	Bind to ACGT Box	Positive	Apple	MdMYB114	Jiang et al. (2021)
MdMYB9/12	Bind to MBS motif	Positive	Apple	MdMYBA1	Wang N. et al. (2018)
MdWRKY11	Bind to W-box	Positive	Apple	MdMYB10/11/12	Liu W. et al. (2019)
MdWRKY72	Bind to W-box	Positive	Apple	MdMYB1	Hu et al. (2020)
MdEIL1	Bind to ATGTA element	Positive	Apple	MdMYB1	An et al. (2018)
AtEIN3	Bind to the EIN3 binding site (TTCAAA)	Negative	*Arabidopsis*	AtPAP1	Meng et al. (2020)
AtGL2	Bind to L1-box	Negative	*Arabidopsis*	AtMYB113, AtPAP2	Wang X. et al. (2015)
AtMYC2	Bind to G-box	Positive	*Arabidopsis*	AtPAP1	Sasaki-Sekimoto et al. (2013)
AtJAM1/2/3	Bind to G-box	Negative	*Arabidopsis*	AtPAP1	Sasaki-Sekimoto et al. (2013)
CBFs	Bind to CBF/DREB elements and interact with MYBs	Positive	Apple	MdMYB12/22/114	Song et al. (2019)
			Eggplant	SmMYB113	Zhou L. et al. (2020)
MdbHLH33	Bind to LTR element and form MBW complex	Positive	Apple	MdMYBA1, MdMYB10	Espley et al. (2007) and Wang N. et al. (2018)
MdbHLH3	Bind to G-box and form MBW complex	Positive	Apple	MdMYB9/11	An et al. (2015)
AtPIF4	Bind to G-box and interact with AtPAP1	Negative	*Arabidopsis*	AtPAP1	Liu Y. et al. (2019)
AtSPX4	Interact with AtPAP1	Negative	*Arabidopsis*	AtPAP1	He et al. (2021)
SlJAF13	Promote *SlAN2-like* expression and form MBW complex	Positive	Tomato	SlAN2-like	Chen et al. (2022)
AtJAZ1	Interact with MYBs and bHLHs to destabilize MBW complex	Negative	*Arabidopsis*	AtPAP1	Qi et al. (2011)
MdBBX37	Interact with MdMYBs	Negative	Apple	MdMYB1, MdMYB9	An et al. (2020c)
MdbZIP44	Interact with MdMYB1	Positive	Apple	MdMYB1	An et al. (2020a)
MdTCP46	Interact with MdMYB1	Positive	Apple	MdMYB1	An et al. (2020a)
MdERF38	Interact with MdMYB1	Positive	Apple	MdMYB1	An et al. (2020b)
Pp4ERF24/Pp12ERF96	Interact with PpMYB114	Positive	Pear	PpMYB114	Ni et al. (2019)
E3 ligases and protein kinases					
COP1	Degradation of MYBs and other upstream light responsive TFs	Negative	*Arabidopsis*	AtPAP1, AtPAP2	Maier et al. (2013)
			Apple	MdMYB1	Li et al. (2012)
MdBT2	Degradation of MdMYB1 and other upstream regulators	Negative	Apple	MdMYB1	Wang X. et al. (2018)
MdSIZ1	SUMOylation of MdMYB1	Positive	Apple	MdMYB1	Zhou et al. (2017)
AtMPK4	Phosphorylation and stabilization of AtPAP1	Positive	*Arabidopsis*	AtPAP1	Li et al. (2016)
Epigenetic regulators					
AtHAT1	Interact with AtPAP1 and deacetylate ABGs promoters	Negative	*Arabidopsis*	AtPAP1	Zheng et al. (2019)
MdAGO4	Methylation of MdMYB1	Negative	Apple	MdMYB1	Jiang et al. (2020)
MdROS1	Demethylation of MdMYB1	Positive	Apple	MdMYB1	Yu L. et al. (2022)

Many TF-binding sites in promoters are responsible for various expression patterns of the key R2R3-MYB activators controlled by different types of upstream regulators. The bZIP transcription factor, ELONGATED HYPOCOTYL 5 (HY5) is a positive regulator of anthocyanin biosynthesis in responding to light and not only binds to promoters of ABGs, but also directly enhances gene expression for R2R3-MYB activators by binding to the G-box or ACE-box in the promoter (An et al., 2017; Sun et al., 2020; Wang et al., 2020; Li L. et al., 2021). For example, in tomato, SlHY5 positively regulates the expression of the master regulator *SlAN2-like* by binding to the ACE-box in the *SlAN2-like* promoter (Qiu et al., 2019; Sun et al., 2020). Another bZIP factor, MdbZIP4-like binds to the ACGT Box of *MdMYB114* promoter to enhance *MdMYB114* expression and thus promotes transcription of anthocyanin biosynthetic genes (Jiang et al., 2021). In *Arabidopsis*, the bHLH transcription factor PHYTOCHROME INTERACTING FACTOR 4 (PIF4) also binds to the G-box of the *AtPAP1* promoter and suppresses *AtPAP1* expression, suggesting that HY5 and PIF4 may competitively bind to G-box to fine-tune transcriptional regulation of R2R3-MYB activators (Liu Z. et al., 2021). In some cases, regulation of R2R3-MYBs is involved in positive or negative feedback loops for fine control of anthocyanin accumulation. The ethylene-responsive gene EIN3-LIKE1 (*EIL1*) directly binds to the promoter of *MdMYB1* and transcriptionally activates *MdMYB1* expression. Conversely, MdMYB1 activates ETHYLENE RESPONSE FACTOR3 (*MdERF3*) expression to enhance ethylene production and *MdEIL1* expression, which results in apple fruit coloration (An et al., 2018). In contrast, the HD-ZIP TF GLABRA2 (GL2), of which expression is induced by the MBW complex, directly represses the expression of *AtPAP2* and *AtMYB113* by binding to the L1-box sequence TAAATGTT/A in their promoters, forming a negative feedback loop for anthocyanin biosynthesis in *Arabidopsis* (Wang X. et al., 2015).

Notably, several TFs are parts of a hierarchical regulatory network that regulates transcript levels of anthocyanin-related R2R3-MYB activators. In apple, the WRKY TF MdWRKY11 promotes anthocyanin biosynthesis by activating *MdMYB10* and *MdMYB11*, which are positive regulators of anthocyanin synthesis in apple flesh, through the binding W-box *cis*-elements in the promoters of the two genes (Liu W. et al., 2019). In addition, MdWRKY11 directly enhances *MdHY5* expression, which also contributes to the upregulation of these R2R3-MYB activators in *MdWRKY11*-overexpressing callus. Similarly, in *MdWRKY72*-overexpressing callus exposed to UV-B irradiation, MdWRKY72 promotes expression level of *MdMYB1* directly by binding the W-box element in the *MdMYB1* promoter and indirectly by binding the W-box element in the *MdHY5* promoter to increase levels of MdHY5, which then enhances expression of *MdMYB1* (Hu et al., 2020). In *Arabidopsis*, the SPX domain protein AtSPX4 indirectly represses expression of *AtPAP1* by interacting with the positive regulator of the phosphate (Pi) starvation signal PHOSPHATE STARVATION RESPONSE1 (PHR1), thus preventing PHR1 from binding to the *cis*-element P1BS in the *AtPAP1* promoter. On the other hand, AtSPX4 physically interacts with AtPAP1, preventing AtPAP1 from binding to the promoter of its target gene (He et al., 2021). Thus, the suppression of AtPHR1and AtPAP1 are released from AtSPX4 during Pi starvation, which promotes *AtPAP1* expression and Pi starvation-induced anthocyanin biosynthesis. Many upstream TFs other than AtSPX4, such as AtLBD37/38/39, AtANAC032 and JUNGBRUNNEN1 (AtJUB1) in *Arabidopsis*, MdLBD13 and MdLOB52 in apple, and BoNAC019 in *Brassica oleracea*, fail to interact with the promoter of R2R3-MYB activators, suggesting the involvement of an indirect regulatory mechanism of anthocyanin accumulation (Rubin et al., 2009; Mahmood et al., 2016; Li H. et al., 2017). The functional mechanism of these repressors in downregulating R2R3-MYB activators needs to be elucidated.

### Natural variations in promoters

Selection pressure during domestication generates phenotypic variations in plants. Notably, natural color variations that have been studied in pigmented tissues in edible, cultivated plants are mainly due to changes in the activity of key R2R3-MYB activators. For instance, mutations in the CDS region of the R2R3-MYB activator can lead to a nonfunctional, truncated protein or a protein with sharply weakened activity that influences coloration in horticultural plants (Castillejo et al., 2020; Sun et al., 2020). In peaches, a deletion in the promoter of *PpMYB10.1* contributes to the upregulation of *PpMYB10.1* and is responsible for the flesh color around the stone, a trait selected during domestication (Guo et al., 2020). The reason of natural color variations mostly mediated by R2R3-MYB activators are possibly due to the multifunctional nature of WDR and bHLHs during plant development and growth, while mutations in these regulators may be extremely deleterious to growth and development and were thus not targets during domestication (Zhang et al., 2003; Baudry et al., 2006; Huang et al., 2019). Compared to WDR and bHLH TFs, SG6 R2R3-MYBs have relatively restricted functions in regulating coloration of pigmented organs that were targeted for selection.

**Figure 1:**
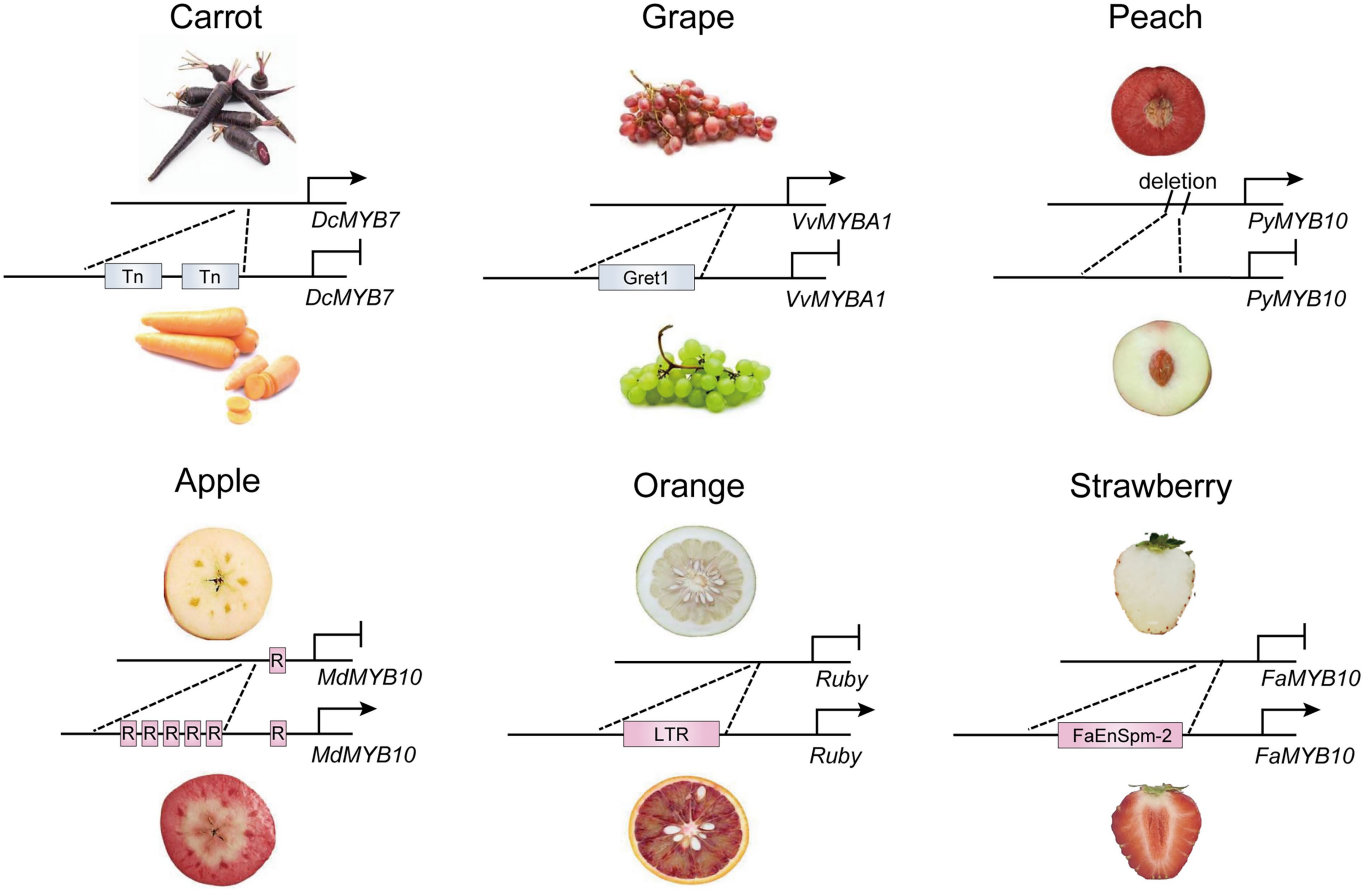
Transcriptional regulation of R2R3-MYB activators by natural variations in promoters. The natural variations in promoters of *DcMYB7* and *FaMYB10* in carrot (Xu et al., 2019) and strawberry (Castillejo et al., 2020) caused by insertion of transposons. In orange (Butelli et al., 2012) and grape (Kobayashi et al., 2004), insertion of retrotransposon leads to variations in promoter of *Ruby* and *VvMYBA1*. The short tandem repeats insertion and fragment deletion have also been found in promoter of *MdMYB10* in apple (Espley et al., 2009) and *PyMYB10* in peach (Guo et al., 2020), respectively. Tn, transposon; Gret1, grapevine retrotransposons 1; R, short tandem repeats; LTR, long terminal repeats; FaEnspm-2, CACTA-like transposon FaEnspm-2; →, activation; —|, repression.

Variations in the promoter region caused by insertion of transposons or fragments alter gene expression by different mechanisms, leading to activation or inactivation of R2R3-MYB activators (Figure 1). In some cases, a transposon insertion causes the inactivation of target genes by changing the structure of the promoter, which prevents the binding of the transcription factor and thus transcription initiation. In carrots, the high expression of *DcMYB7* in purple root cultivars, not variations in the coding region, contributes to root pigmentation. A nonfunctional tandem duplication and two transposon insertions in the promoter of *DcMYB7* lead to the transcriptional inactivation of *DcMYB7* in nonpurple carrot roots (Xu et al., 2019). Similarly, the insertion of *Gret1* (*Grapevine retrotransposon 1*) has been found in the upstream region of *VvMYBA1* in white grape cultivars, which disrupts transcription of *VvMYBA1* in grape skin (Kobayashi et al., 2004). On the contrary, insertion of some transposable elements in the genomic region may activate expression of an adjacent gene, because transcription factor binding sites or transcription start sites are usually found in the insertion fragments. A *copia*-like retrotransposon in the promoter of *Ruby* activates its expression in the flesh of blood oranges by providing *cis*-elements in the LTR region (Butelli et al., 2012). Also, in pepper, the expression of *CaAN2* is activated by insertion of a 4.2-kb non-long terminal repeat (non-LTR) retrotransposon in the purple cultivar KC00134, which has been predicted to contain known transcription-factor-binding sites for anthocyanin regulators (Jung et al., 2019). Natural color variations in strawberry fruit are main controlled by FaMYB10. In octoploid cultivated strawberry, a CACTA-like transposon (FaEnSpm-2) insertion in the *FaMYB10-2* promoter enhances the expression of *FaMYB10-2* in red-fleshed lines, but the *cis*-elements responsible for enhanced *FaMYB10-2* expression in FaEnSpm-2 remain to be characterized (Castillejo et al., 2020). However, to date, identifying upstream regulators that directly bind to the insertion LTR regions has been difficult because an unexpected association may occur with an unknown TF(s) or epigenetic regulator(s) in multiple predicted binding sites of the promoter.

Several lines of evidence have shown that multiple short tandem repeats in the promoter of R2R3-MYB activators enhance transcriptional activity of the activator itself. In one case, the number of tandem repeats in the *MdMYB10* promoter is associated with the targeted self-activation of *MdMYB10* to form a positive feedback loop for anthocyanin accumulation in red-fleshed apple varieties (Espley et al., 2009). Similarly, *Red Leaf Cotton 1* (*RLC1*), the ortholog of *AtPAP1*, is highly expressed in red leaf cotton, and its promoter contains more tandem repeats than in green leaf cotton, which leads to differences in the expression level of *RLC1* and thus in the leaf color between the two cultivars (Gao et al., 2013).

### Epigenetic and posttranscriptional modifications

Epigenetic modifications such as DNA methylation and demethylation, histone modifications and microRNAs (miRNAs) play important roles in plant development and growth by enhancing the plasticity of chromatin and regulating gene expression. Heritable DNA methylation in the promoter of R2R3-MYB transcriptional activators has also been shown to be associated with anthocyanin accumulation (Sekhon and Chopra, 2009; Qian et al., 2014; Jiang et al., 2020). The pear cv. Zaosu Red, a bud sport of cv. Zaosu, has red shoots, red young leaves, and fully red or striped fruit. The DNA methylation levels of the *PyMYB10* promoter are lower in anthocyanin-rich tissues (Qian et al., 2014). Similarly, analysis of the dynamics of global DNA methylation in apple fruit using whole-genome bisulfite sequencing revealed that hypomethylation of the promoters of flavonoid biosynthetic genes and *MdMYB10* leads to anthocyanin accumulation in the fruit skin (Li et al., 2019). Recently, the DNA methylation mechanism of R2R3-MYB activators in anthocyanin biosynthesis has been revealed to be mediated by ARGONAUTE RISC COMPONENT 4 (AGO4) through RNA-directed DNA methylation (RdDM) pathway (Jiang et al., 2020).

**Figure 2:**
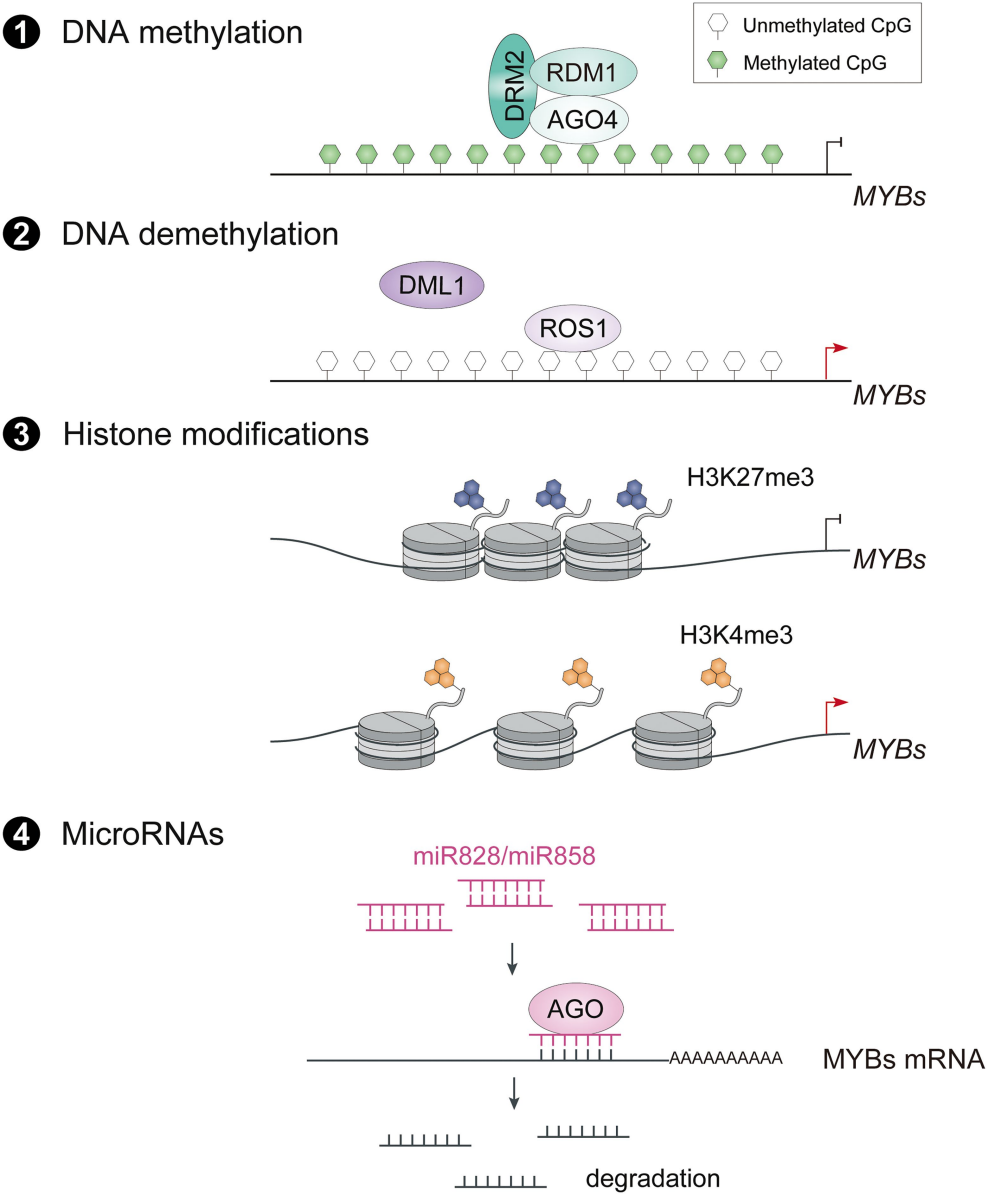
Epigenetic and posttranscriptional regulation on expression of R2R3-MYB activators. The transcript level of R2R3-MYB activators is also regulated through AGO4 mediated RNA-directed DNA methylation (RdDM) pathway (1), ROS1 mediated DNA demethylation (2), Histone modifications (3) and miR828/miR858 mediated degradation of mRNAs (4). ROS1, repressor of silencing 1; DML1, demeter-like 1; H3K27me3, trimethylation at lysine 27 of histone H3, H3K4me3, trimethylation at lysine 4 of histone H3; AGO, argonaute.

The dynamic regulation of DNA methylation in the promoter region of R2R3-MYB activators enables plants to adapt to environmental changes (Figure 2). In blood oranges during cold storage, decreased cytosine methylation levels of the *Ruby* promoter result in a highly pigmented region in the fruits by activating *Ruby* expression (Sicilia et al., 2020). In particular, the cold temperature induces production of DNA demethylase DEMETER-LIKE1 (DML1), which may contribute to higher expression of *Ruby* in response to cold stress (Sicilia et al., 2020). Similarly, REPRESSOR OF SILENCING 1 (ROS1), which encodes a DNA glycosylase DEMETER (DME) family protein, promotes demethylation of *MdMYB10* in apple leaves at low temperature, demonstrating that demethylation of R2R3-MYBs is involved in anthocyanin accumulation induced by low temperature (Yu L. et al., 2022). Some fruits such as apple and pear are placed in paper bags to accumulate more anthocyanins in their fruit skin after fruits are removed from the bags (Bai et al., 2017; Cho et al., 2022). This accumulation in apples is associated with epigenetic changes in the promoter of *MdMYB10* and *MdMYB1*. The bag removal treatment causes demethylation of *MdMYB10*, which promotes a blush color in the fruit skin, in part due to the downregulated expression of genes for DNA methyltransferases such as MdMET, MdCMT, and MdDRM (Cho et al., 2022). The CG-type methylation level of the *MdMYB10* promoter in the blushed skin was significantly lower than in red-striped skin, indicating the importance of differential DNA methylation levels in the promoter regions of key MYB transcription factors on stripe phenotypes (Jia et al., 2020). In white light + UV-B, the level of the active histone marker H3K4me3 is higher in the *MdMYB1* promoter and that of the inactive histone marker H3K27me3 is lower immediately after bag removal (Bai et al., 2016). Therefore, the darkness of the bagging treatment influences the epigenetic status of R2R3-MYB activators, and exposure to light after bag removal releases the binding sites of upstream TFs to activate their expression and thus anthocyanin accumulation. However, no bagged fruits or bag removal fruits at early developmental stage accumulate less anthocyanins, indicating that a developmental signal may promote turnover of the epigenetic status of fruit skin from hyposensitive to hypersensitive to light during bagging treatment.

MicroRNAs (miRNAs) are involved in epigenetic regulation by modulating R2R3-MYBs posttranscriptionally. Evidence is accumulating that miRNAs are involved in anthocyanin accumulation by targeting anthocyanin-related R2R3-MYBs (Figure 2). For example, miR828 and miR858 target multiple anthocyanin-related MYB activators and repressors, and trigger the cleavage of their transcripts, indicating dual regulatory roles in anthocyanin biosynthesis (Jia et al., 2015; Wang et al., 2016; Bonar et al., 2018; Zhou B. et al., 2020). In *Arabidopsis*, miR828 directly targets the highly conserved R3 domain of AtMYB113 and reduces its transcript level (Luo et al., 2012). On the other hand, miR828 also indirectly degrades transcripts of *AtMYB113*, *AtPAP1* and *AtPAP2* by producing siR81(−) from the cleavage of transcripts of *Trans-Acting SiRNA Gene 4* (*TAS4*; Luo et al., 2012). Similarly, miR858 targets a cleavage site in SlMYB7-like, a positive regulator of anthocyanin biosynthesis in tomato, and thus negatively regulates anthocyanin production (Jia et al., 2015). Notably, the abundance of miR858 and miR828 is regulated by light in a different manner depending on the species, suggesting that posttranscriptional regulation of R2R3-MYB activators by miRNAs is involved in light-induced anthocyanin accumulation.

## Regulation of R2R3-MYB activators at the protein level

**Figure 3:**
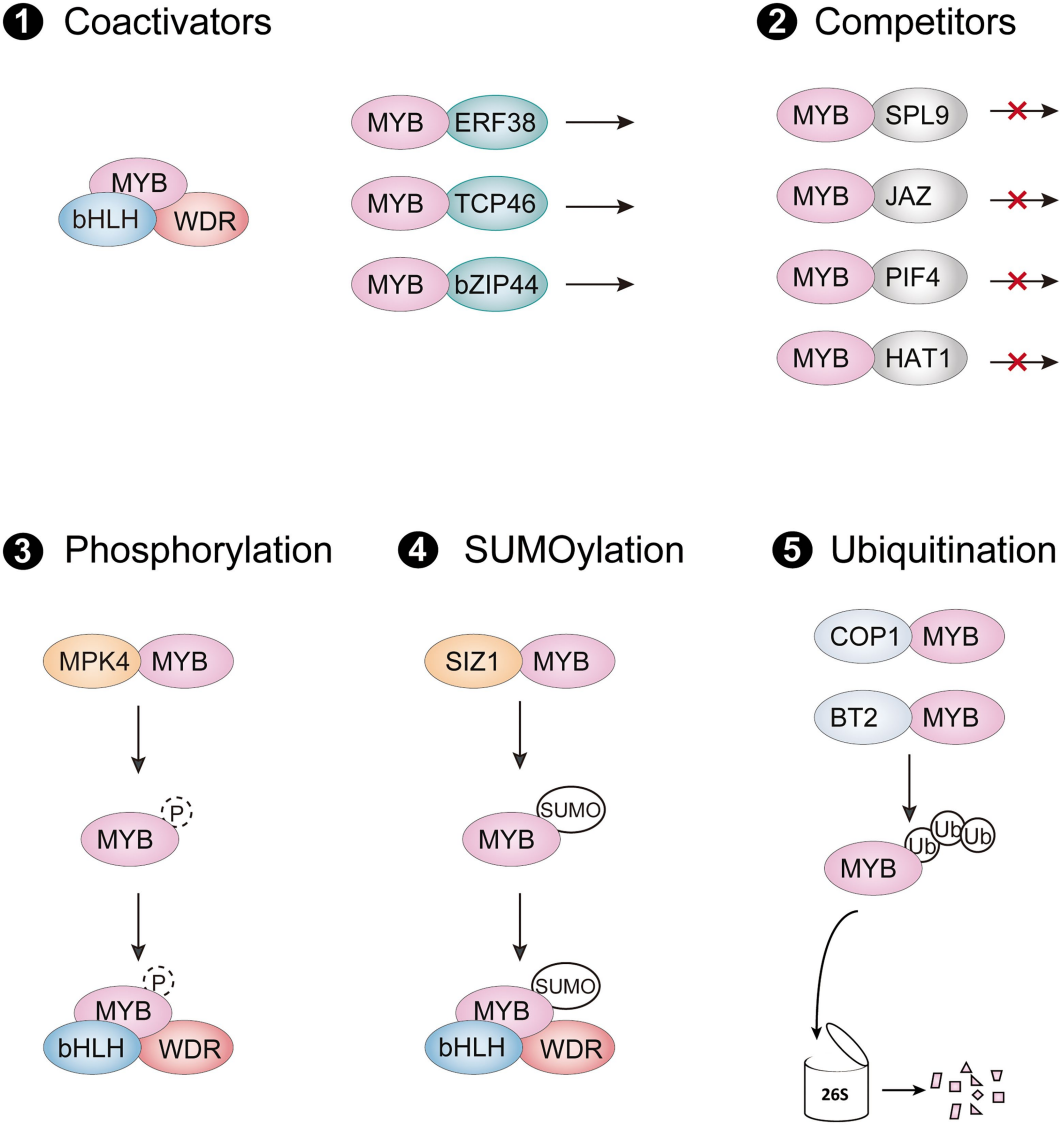
Posttranslational regulation of R2R3-MYB activators. (1) R2R3-MYBs form an active MBW complex with bHLHs and WDR protein, or they interact with other coactivators to promote their transcriptional activity. (2) The interaction of R2R3-MYB activator with competitors interferes stabilization of the MBW complex and represses their activation of anthocyanin biosynthetic genes (3–5). R2R3-MYB activators can be regulated through posttranslational modifications such as phosphorylation (3), SUMOylation (4), and ubiquitination (5). SUMO, small ubiquitin-like modifier; Ub, ubiquitin molecules.

In the last decade, many MYB-interacting proteins (MIPs) have been shown to modulate protein activity of R2R3-MYB activators through protein–protein interactions. These MIPs include different types of transcription factors, E3 ligases, and protein kinases, which are involved in multiple signaling pathways to regulate anthocyanin accumulation by targeting R2R3-MYB activators in different environments (Table 1). Regulation of R2R3-MYB transcriptional activators at the protein level is critical for precise control of their activity, mainly through five routes: (i) forming an active MBW complex with R2R3-MYBs; (ii) interfering with the formation of MBW complex by interacting with R2R3-MYBs; (iii) enhancing the activity of R2R3-MYBs for activating downstream genes; (iv) destabilization of R2R3-MYBs by degradation; (v) stabilization of R2R3-MYBs by phosphorylation or SUMOylation (Figure 3).

### Interaction with coactivators or competitors

As a master regulatory complex, the MBW complex activates numerous ABGs in all angiosperms studied so far. In *Arabidopsis*, subgroup 6 R2R3-MYB activators promote anthocyanin accumulation by interacting with IIIf bHLH members TESTA GLABRA 8 (TT8), ENHANCER of GLABRA 3 (EGL3) and GLABRA 3 (GL3) *via* the bHLH-interacting motif in their R3 MYB domain, which also exists in several R3 MYB and subgroup 4 R2R3-MYB repressors (Ma and Constabel, 2019). The bHLH-interacting motif is important for the protein-binding function of these R2R3-MYB activators, since mutations at certain amino acids in the motif lead to a large reduction in anthocyanin accumulation (Grotewold et al., 2000). However, several amino acids, apart from the bHLH interaction motif, also influence the interaction with bHLH partners, probably by altering the R2R3-MYB protein conformation (Hichri et al., 2011; Zhou et al., 2018; Yang et al., 2021). Although these bHLHs belong to the same subgroup, they are grouped into two subclades and have different roles in some species (Albert et al., 2014; Chen et al., 2022). The MBW complex formed by the two bHLHs subclades has a hierarchical regulatory mechanism, in which the upstream bHLHs activate the expression of downstream bHLHs by forming the MBW complex, but amino acid variations between these bHLHs do not affect the interaction with anthocyanin-related R2R3-MYB proteins (Zhang et al., 2019). Notably, several bHLHs have been proved to regulate expression of their MYB partners for stabilization of MBW complex. For instance, MdbHLH3 and MdbHLH33 not only form active MBW complex with MdMYBs, also activate their expression (An et al., 2015; Wang N. et al., 2018). Similarly, the bHLH TF SlJAF13, component of MBW complex in tomato, promotes expression of *SlAN2-like* gene at the transcriptional level (Chen et al., 2022). However, since *SlAN2-like* is not regulated by the MBW complex or SlJAF13 directly, it is reasonable to speculate that yet-to-be identified co-factors regulate *SlAN2-like* expression by interacting with SlJAF13. The other component of the MBW complex, the WDR protein, commonly acts as a bridge for R2R3-MYBs and bHLHs to enhance stabilization of the transcriptional activation complex (Zhang et al., 2003). To date, numerous bHLHs and WDR proteins, that are homologs of AtTT8, AtEGL3 and AtTTG1, have been identified in many plants and found to work similarly to those in *Arabidopsis*.

Other MIPs have also recently been discovered to interact with R2R3-MYB activators and modulate transcriptional activity of R2R3-MYBs to anthocyanin biosynthetic genes. Two ethylene response factors (ERF), Pp4ERF24 and Pp12ERF96, promote red coloration of pear fruit by directly interacting with PpMYB114 and enhancing PpMYB114-mediated *PpUFGT* expression (Ni et al., 2019). Similarly, MdERF38 promotes drought-stress-mediated anthocyanin biosynthesis in apple by enhancing the binding activity of MdMYB1 to *MdDFR* and *MdUF3GT* (An et al., 2020b). Other regulators such as the TCP protein MdTCP46, bZIP protein MdbZIP44 and WRKY protein MdWRKY40 promote anthocyanin biosynthesis in response to multiple stimuli by facilitating the transcriptional activity of MdMYB1 (An et al., 2020a). In contrast to MdERF38, the proteins MdBBX37 and AtSPX4 negatively regulate anthocyanin biosynthesis by interfering with the respective binding activities of MdMYB1 and AtPAP1 to the promoter of ABGs, respectively (An et al., 2020c; He et al., 2021).

Also, many anthocyanin repressors interfere with anthocyanin biosynthesis by interacting with R2R3-MYB transcriptional activators, disrupting the formation of MBW complex or changing the epigenetic status of downstream biosynthetic genes (Figure 3). In *Arabidopsis*, the miR156-targeted SQUAMOSA PROMOTER BINDING PROTEIN-LIKE9 (SPL9), jasmonate-ZIM-domain proteins (JAZs), and PIF4 are found to directly interact with AtPAP1, impairing the transcriptional activity of the MBW complex (Gou et al., 2011; Qi et al., 2011; Qin et al., 2022). A class II HD-Zip protein AtHAT1 recruited the TOPLESS corepressor *via* its EAR motifs in the N terminal, which forms a transcriptional repressor complex with AtPAP1 to destabilize the MBW complex and suppress histone H3 acetylation of ABG promoters (Zheng et al., 2019). In maize, the epigenetic regulator R-INTERACTING FACTOR 1 (RIF1) forms a transcriptional activation complex with the bHLH protein R and MYB protein C1, two components of the MBW complex, and thus promotes acetylation of H3K9 and H3K14 in the promoter of ABGs (Hernandez et al., 2007). The interaction of epigenetic regulators with R2R3-MYBs or bHLHs of the MBW complex recruit histone modification enzymes to facilitate epigenetic changes of the ABGs and contribute to precise regulation of anthocyanin accumulation.

### Posttranslational modifications

Posttranslational modifications (PTMs) of proteins are mediated by enzymes that catalyze reactions with the target protein, thereby modulating the activity and abundance of the protein in certain conditions. Ubiquitination, SUMOylation and phosphorylation are common PTMs that regulate protein function and abundance by modifying protein structure. Several enzymes that mediate anthocyanin accumulation by regulating PTMs of R2R3-MYB activators have been discovered, improving our understanding of PTMs that affect anthocyanin production.

#### Ubiquitination by E3 ligases

Ubiquitination is the covalent conjugation of ubiquitin to target proteins by an E3 ligase, which promotes the degradation of the protein through the 26S proteasome. Some E3 ligases have been reported to interact with R2R3-MYB transcriptional activators and mediate their degradation, leading to the suppression of anthocyanin accumulation. In *Arabidopsis*, E3 ligase CONSTITUTIVE PHOTOMORPHOGENIC1 (COP1) acts as a negative regulator in light signaling by degrading AtHY5 proteins, the upstream regulator of the MBW complex. The key R2R3-MYB regulators of anthocyanin biosynthesis are also directly targeted and degraded by COP1, resulting in anthocyanin deficiency in the dark (Li et al., 2012; Maier et al., 2013). In addition, the E3 ligase BROAD-COMPLEX, TRAMTRACK AND BRIC A BRAC2 (MdBT2) regulates anthocyanin biosynthesis in response to nitrate application by promoting ubiquitination of MdMYB1 (Wang X. et al., 2018). Similar to the action of COP1, MdBT2 also inhibits upstream regulators of MdMYB1 to control anthocyanin accumulation in response to various environmental signals. For instance, MdBT2 disrupts MdBBX-MdHY5 complex activity by degrading MdBBX22, thus preventing transcriptional activation of *MdMYB10* in response to UV-B irradiation (An et al., 2019). MdERF38, MdTCP46, MdbZIP44 and MdWRKY40 have also been found to be coactivators of MdMYB1; they act as ubiquitination targets of MdBT2, indicating that MdBT2 might be a central component for anthocyanin accumulation (An et al., 2020a). MdBT2 was also recently demonstrated to promote the stabilization of MdCOP1 in the dark and the degradation of MdMYB1 by MdCOP1 (Kang et al., 2022). In addition, suppression of MdCOP1 in MdBT2-overexpressing leaves revealed anthocyanin levels that were as high as in MdCOP1-suppressed leaves, suggesting that MdCOP1 is required for the function of MdBT2 and has a dominant role in the degradation of anthocyanin regulators during exposure to light **+** UV-B. Because many anthocyanin regulators are targeted by both MdBT2 and MdCOP1, these two E3 ligases MdBT2 and MdCOP1 might also coordinately regulate protein levels of other positive regulators such as MdHY5 and several MdBBXs to repress anthocyanin biosynthesis in certain conditions.

#### Phosphorylation by MPKs

Mitogen-activated protein kinase (MAPK) cascades have been implicated in multiple stress signal transduction pathways. The downstream MAP kinases (MPK) are phosphorylated and activated by upstream MAP kinase kinases (MKK), which subsequently phosphorylate and thus activate R2R3-MYB activators to promote anthocyanin accumulation in *Arabidopsis*. Phosphorylation of MYB75 at T131 and T126 by MITOGEN-ACTIVATED PROTEIN 4 (MPK4) promotes stabilization of MYB75 and thus anthocyanin accumulation in light (Li et al., 2016). Moreover, the stability of MYB75 increases when it is co-expressed with active MKK1, the MAPK kinase that acts upstream of MPK4. In contrast to MKK1, the upstream MAPK kinase MKK6 negatively regulates anthocyanin accumulation by suppressing MYB75 expression independently of MPK4, indicating that additional MPKs may be involved in the process (Wersch et al., 2018). MYB75 can also be phosphorylated primarily at T131 by other multiple MAP kinases (i.e., MPK3, MPK6, and MPK11), leading to the hypothesis that MYB75 may be regulated by multiple signaling pathways as a common target of multiple MAP kinases (Kreynes et al., 2020). However, phosphomimic MYB75 generated by T131E does not affect anthocyanin accumulation in MYB75^T131E^-overexpressing plants compared to the wild-type plants, which is consistent with the lack of effect of MPK3 and MPK6 on light-induced anthocyanin accumulation. Thus, the effects on phosphorylation of MYB75 by other MAP kinases need to be further elucidated.

#### SUMOylation by small ubiquitin-like modifier E3 ligases

SUMOylation has been shown to be a major mechanism for regulating protein stability and function during plant development and stress responses. Similar to the case of ubiquitin, the covalent conjugation of SUMO (small ubiquitin-like modifier) requires the interaction of specific SUMO E3 ligases with their targets. The small ubiquitin-like modifier E3 ligase SIZ1, which is a multifunctional protein, mediates SUMOylation of multiple proteins that are involved in Pi deficiency, shoot regeneration, aluminum resistance, and plant immunity (Zhou et al., 2017; Niu et al., 2019; Xu et al., 2021). In *Arabidopsis thaliana*, mutations in AtSIZ1 lead to severe growth deficiencies and less anthocyanin accumulation. The transcript levels of *AtPAP1*, *AtTT8* and core ABGs during drought are lower in *siz1-3* mutants, indicating that SUMO modification mediated by AtSIZ1 promotes anthocyanin accumulation under drought stress (Catala et al., 2007). SIZ1 is also a potential integrator in response to environmental changes, since expression of SIZ1 is induced by multiple signals (e.g., cold, salt, drought, Pi deficiency, and abscisic acid; Zhang et al., 2017; Jiang et al., 2022). At low temperature, the SUMOylation of MdMYB1 by MdSIZ1 stabilizes MdMYB1 and promote anthocyanin accumulation in apple fruit and callus (Zhou et al., 2017). Recently, the MYB transcription factor MdMYB2 was proven to activate MdSIZ1 in response to cold and thus promote anthocyanin accumulation by MdSIZ1-mediated SUMOylation of MdMYB1 (Jiang et al., 2022). Characterization of other regulators that induce SIZ1 expression and other substrates that are targeted by SIZ1 should help us to better understand SUMOylation effects on anthocyanin production in various conditions.

## R2R3-MYB is the central hub to integrate different signaling pathways to mediate anthocyanin biosynthesis

The regulation of anthocyanin biosynthesis by R2R3-MYB activators depends on regulators that are involved in light, extreme temperatures, sugar, and hormone signal transduction pathways. Light, low temperature, sugar, and JA induce anthocyanin biosynthesis, while other signals such as high temperature, ethylene and GA tend to negatively affect anthocyanin production (Teng et al., 2005; Qi et al., 2011; Liu et al., 2018; Liu Y. et al., 2019; Kim et al., 2020). Recently, regulatory mechanisms of anthocyanin accumulation that target R2R3-MYB activators have been characterized for these signaling pathways, and a few components that function in the integration of these signals have been identified, providing new insights into how plants regulate anthocyanin production in response to multiple stimulus.

### Light signaling pathway

**Figure 4:**
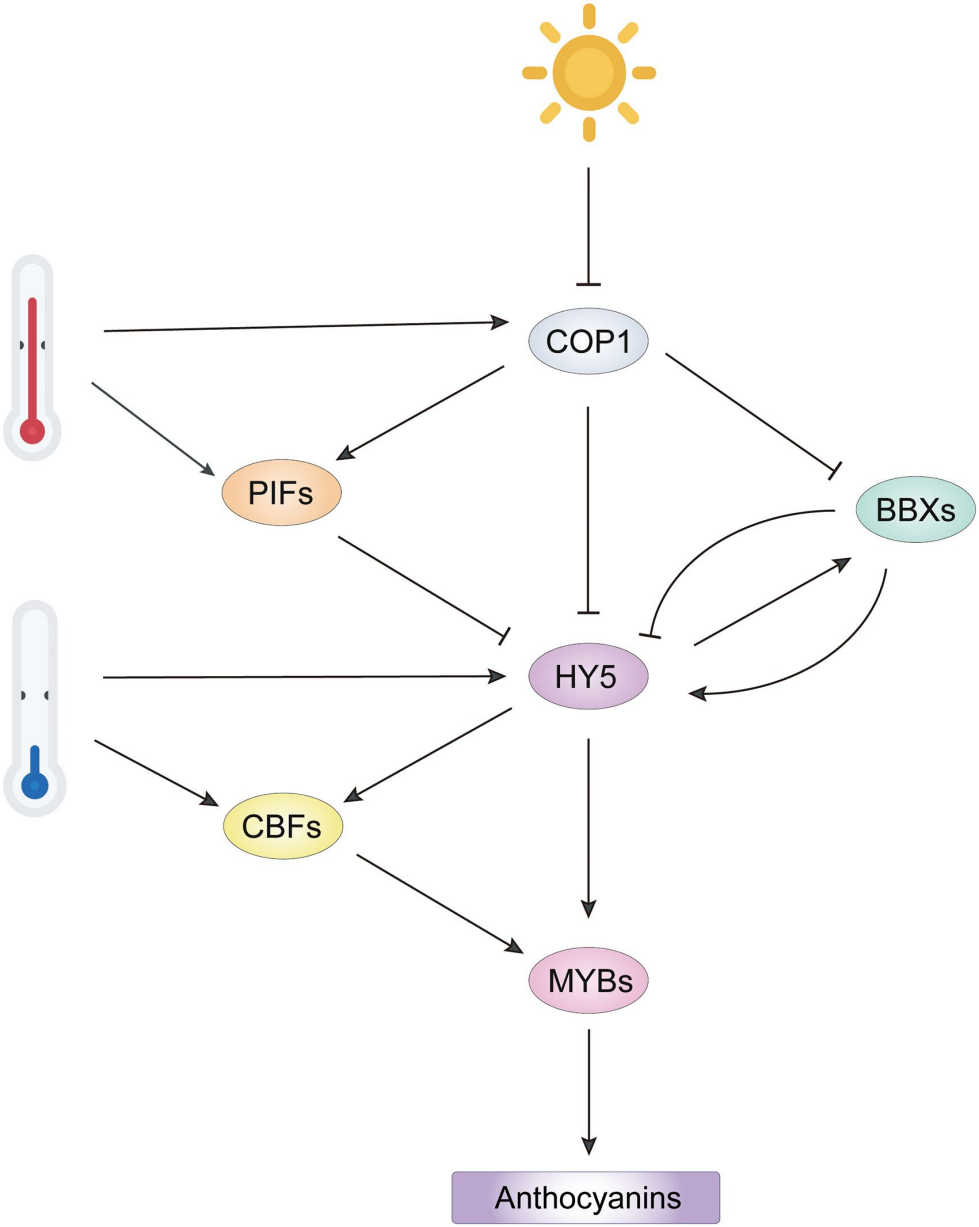
Integrated network of light-and temperature-mediated anthocyanin biosynthesis by regulation of R2R3-MYB activators. Light represses COP1 activity to release HY5 and BBX, promoting formation of an HY5-BBX module to activate downstream R2R3-MYB activators. However, BBX repressors inhibit HY5 activity by interfering the interaction of HY5 with other BBX activators. High temperature represses anthocyanin biosynthesis by stabilizing COP1 and PIFs to inactivate HY5. Low temperature induces HY5 and CBF expression, which promotes transcription of R2R3-MYB activators. The COP1/PIFs-HY5/BBXs-MYB cascade integrates the regulation of R2R3-MYB activators from light and temperature signals to regulate anthocyanin biosynthesis.

The role of light in relation to the regulation of anthocyanin accumulation has been investigated in apple (An et al., 2017; Henry-Kirk et al., 2018), peach (Zhao et al., 2017), pear (Bai et al., 2017), grape (Guan et al., 2016), eggplant (Li J. et al., 2017) and litchi (Zhang et al., 2016). Far red light, red light, blue light, UV-A and UV-B induce anthocyanin accumulation in plants, with short wavelengths (blue light to UV-B radiation) more effective (Zhou et al., 2007; Kim et al., 2020). Although plants have different routes for the perception and transduction of different wavelengths of light, the COP1-HY5 module is a common target of photoreceptors (Figure 4). Light-activated photoreceptors interact with COP1, leading to repression of COP1 function and stabilization of light-responsive factors AtHY5, AtPAP1, AtPAP2, and AtBBXs, and thus to the upregulation of ABGs (Li et al., 2012; Maier et al., 2013; Xu et al., 2016). Recently, several regulators upstream of COP1 have been identified to regulate anthocyanin accumulation in a COP1-dependent manner. In *Arabidopsis*, ANGUSTIFOLIA3 (AN3) acts as a positive regulator of light-induced anthocyanin biosynthesis by repressing *COP1* expression. However, AN3 probably acts as a cofactor with another regulator for binding the *COP1* promoter, since AN3 does not bind to the COP1 promoter *in vitro*, but does *in vivo* (Meng, 2015). In apple, the COP1-interacting proteins (CIPs) MdCIP8 and MdCIP1 inhibit anthocyanin biosynthesis by increasing the activity of MdCOP1, but the mechanism remains unclear (Kang et al., 2020, 2021).

Other bHLH family members, phytochrome-interacting factors (PIFs), are negative regulators in light signaling and stabilized by COP1 in the dark (Luo et al., 2014; Kathare et al., 2020). Normally, PIFs competitively bind to HY5-targeted *cis*-elements in the promoters of anthocyanin-related genes and repress their expression (Liu et al., 2015). However, there are some exceptions. PIF3 and HY5 bind to different regions of anthocyanin biosynthetic gene promoters and enhance anthocyanin accumulation in response to far red light, which is dependent on a functional HY5 (Shin et al., 2007). In the regulation of light-responsive anthocyanin biosynthesis, HY5 and PIFs form a dynamic regulatory module, facilitating the transcription of *AtPAP1* and anthocyanin biosynthetic genes in *Arabidopsis*.

As mentioned above, HY5 positively regulates anthocyanin accumulation in light by activating key R2R3-MYB regulators. The full function of HY5 requires its interaction with BBX family members, which contain two B-box domains (Datta et al., 2008; Bursch et al., 2020). In *Arabidopsis*, at least three BBX proteins BBX22, BBX21 and BBX20 positively regulate anthocyanin biosynthesis in an HY5-dependent manner (Figure 4). They form BBX-HY5 complexes with HY5 to enhance transcription of *AtPAP1* and other ABGs (Chang et al., 2008). In addition, BBX20 and BBX21 directly induce expression of *HY5* (Wei et al., 2016; Xu et al., 2016). In pear, the interaction of PpBBX16 with PpHY5 is required to induce *PpMYB10* expression by formation of the PpHY5-PpBBX16 complex (Bai et al., 2019). In apple, MdBBX20, MdBBX22 and MdBBX33/MdCOL11 have roles similar to that of PpBBX16, suggesting that BBX proteins have redundant roles in the fine regulation of anthocyanin biosynthesis (Bai et al., 2014; An et al., 2019; Fang et al., 2019b). In contrast, BBX25, BBX24 and BBX32 function as negative regulators of anthocyanin biosynthesis by forming inactive heterodimers with HY5 or BBX21, thereby reducing the transcriptional activity of the HY5-BBX complex (Holtan et al., 2011; Gangappa et al., 2013). Transcriptional regulation of anthocyanin biosynthesis by the HY5-BBX module has also been found in rice (Kim et al., 2018) and grape (Liu W. et al., 2021) and presumably is quite conserved in plants.

### Temperature signaling pathway

Low temperature (LT) treatment is a common strategy to improve pigmentation traits in horticultural plants. LT induces gene expression for key positive regulators that form the active MBW complexes to enhance anthocyanin accumulation (Xie et al., 2012; Wang N. et al., 2018; He et al., 2020; Zhou L. et al., 2020). C-REPEAT BINDING FACTOR (CBF) members are central regulators during low temperature signaling and participate in the LT-induced anthocyanin accumulation by regulating transcriptional activity of MBW complex. At LT, gene expression for MYB transcription factors MdMYB12, MdMYB22 and MdMYB114 is highly correlated with anthocyanin accumulation, and these genes have several CBF/DREB response elements in their promoters (Song et al., 2019). In addition, three SmCBFs interact with SmMYB113 and facilitate anthocyanin accumulation in eggplants, indicating that the regulation of R2R3-MYB activators by CBFs is involved at both transcriptional and protein levels (Zhou L. et al., 2020).

Mutations in HY5 and HY5 HOMOLOG (HYH) abolished LT-induced anthocyanin accumulation, indicating the involvement of HY5 on anthocyanin accumulation in response to LT (Zhang et al., 2011). LT induces the expression of HY5 and increases the abundance of HY5 by attenuating the HY5-COP1 interaction through CRYPTOCHROME2 (CRY2), a blue light photoreceptor (Li Y. et al., 2021). Beside direct activation of anthocyanin regulators, HY5 activates the expression of *CBF* genes, which promotes R2R3-MYB activators in an indirect manner at LT (Zhang et al., 2020).

High temperature represses anthocyanin biosynthesis by reducing the expression of key MYB activators. In *Cymbidium* flowers, gene expression for the positive regulator CyMYB1 is suppressed at high temperature (30°C/25°C) compared to its expression at 20°C/15°C (Nakatsuka et al., 2019). In potato, high temperature significantly reduces the expression of *StAN1* and *StbHLH1*, two positive regulators of skin and flesh pigmentation, and induces the expression of *StMYB44-1* and *StMYB44-2*, which encode two R2R3-MYBs that promote lignin biosynthesis, another branch of phenylpropanoid pathway, to reduce anthocyanin biosynthesis (Liu Y. et al., 2019). In *Arabidopsis*, the repression of anthocyanin biosynthesis by high temperature is mainly due to degradation of the HY5 protein in a COP1 activity-dependent manner instead of repression of *HY5* expression (Kim et al., 2017). The increased COP1 activity in high temperature is associated with the positive regulatory role of SUMO E3 ligase SIZ1, which also affects the expression of a substantial subset of PIF4-regulated genes (Hammoudi et al., 2018). In addition, high temperature induces *AtPIF4* expression and stabilizes AtPIF4, which inhibits activity of the MBW complex by targeting AtPAP1 at the transcriptional and protein levels (Proveniers and van Zanten, 2013; Liu Z. et al., 2021; Qin et al., 2022). In apple, two heat shock transcription factors (HSFs) MdHSF3b and MdHSF4a activate *MdCOL4* expression at high temperature, causing the inactivation of MdHY5 through the formation of the MdHY5-MdCOL4 complex (Fang et al., 2019a). Collectively, the COP1/PIFs-HY5/BBXs-MYBs signaling cascades thus act as critical integratory module of transduction signals from light and temperature to optimize anthocyanin biosynthesis in plants.

### Sugar signaling pathway

In *Arabidopsis*, sucrose, maltose and glucose can elevate anthocyanin levels. Sugar-dependent anthocyanin accumulation is mediated by sucrose transporters (SUCs) or proteins closely associated with SUCs to modulate endogenous soluble sugars levels and induce a signaling pathway (Lloyd and Zakhleniuk, 2004; Sivitz et al., 2008). Mutations in SUC1 leads to decreased anthocyanin accumulation in seedlings that are grown in 3% w/v sucrose (Sivitz et al., 2008), and the soluble sugar content (glucose, fructose and sucrose) in the *suc1-2* mutant is 56% lower than in the wild type, indicating that sucrose uptake by SUC1 is necessary for sucrose-induced anthocyanin production (Jeong et al., 2010). Similarly, another NAC TF ANAC032 is induced by sucrose in a concentration-dependent manner and acts as a negative regulator of anthocyanin accumulation in part by altering sugar metabolism (Mahmood et al., 2016).

**Figure 5:**
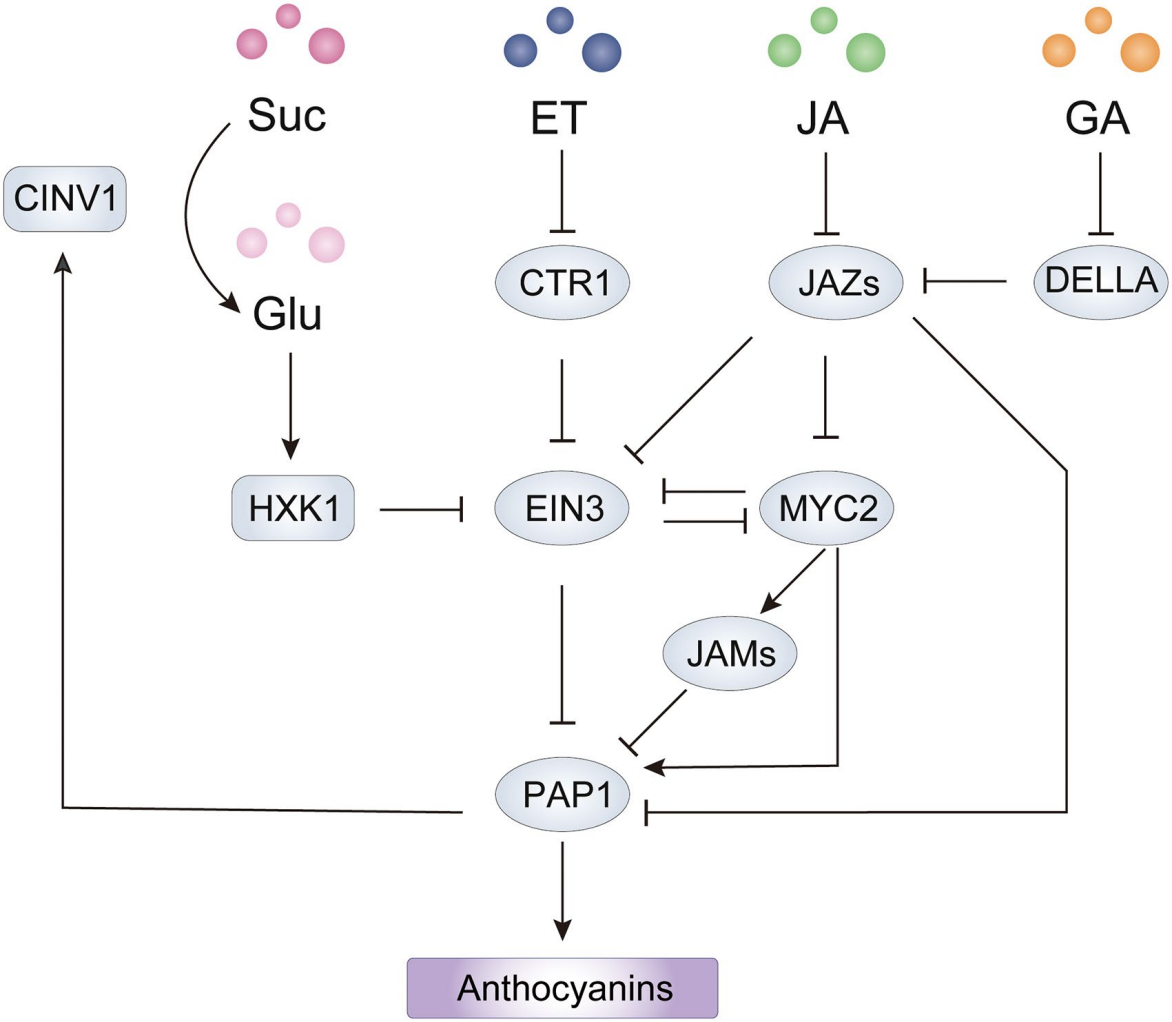
Integrated network of sugar and hormones involved in anthocyanin biosynthesis mediated by PAP1 in *Arabidopsis*. The HXK1-EIN3-PAP1 cascade forms a positive feedback loop for sugar induced anthocyanin biosynthesis. The point(s) of convergence of EIN3-dependent sucrose, ET, and JA signaling pathways lead to transcriptional regulation of PAP1. The JAZ-MYC2 module acts as an integrated node of ET, GA and JA signaling pathways for regulation of PAP1 at the transcriptional and translational level. HXK1, hexokinase 1; CINV1, cytosolic invertase 1; ET, ethylene; JA, jasmonate; GA, gibberellin; Suc, sucrose; Glu, glucose.

Sugar signaling generally regulates the TFs associated with anthocyanin biosynthesis at transcriptional levels (Figure 5). In *Arabidopsis*, exogenous sucrose induces expression levels of anthocyanin biosynthetic genes through the upregulation of positive TFs such as AtGL3, AtTT8 and AtPAP1 (Teng et al., 2005; Jeong et al., 2010). Natural variations in *AtPAP1/MYB75* determine anthocyanin levels in response to sucrose in different ecotypes (Teng et al., 2005), while a 90-bp intronic sequence in *AtPAP1* functions as a sucrose response element that is responsible for regulating *AtPAP1* expression (Broeckling et al., 2016). Two recent studies on *A. thaliana* showed that the regulator ETHYLENE-INSENSITIVE3 (EIN3) functions as a negative regulator in sugar-mediated signaling pathway. Expression, biochemical and genetic analyses showed that EIN3 can interact with YODA (YDA), a MAP kinase kinase kinase and directly bind to the promoter of *AtTT8* and *AtPAP1*, thereby repressing their expression (Meng et al., 2018, 2020). In addition, genetic analysis revealed that AN3 is an upstream negative regulator of YDA and a positive regulator of anthocyanin biosynthesis, but the mechanism by which the AN3-YDA module regulates sucrose levels is unknown (Meng et al., 2016).

Because sucrose itself is not the signaling molecule, sucrose needs to be reversibly hydrolyzed by invertase, yielding glucose to trigger sugar signaling and responses in plants (Sheen et al., 1999). The action of invertase provides glucose for sucrose-dependent anthocyanin biosynthesis by catalyzing sucrose catabolism, which is regulated by R2R3-MYB activators (Payyavula et al., 2013; Meng et al., 2020). Interestingly, in many accessions of *A. thaliana*, exogenous sucrose can induce anthocyanin accumulation at higher levels than other sugars can, by inducing *PAP1* expression, then PAP1 binds directly to the promoter of *CYTOSOLIC INVERTASE1* (*CINV1*) to initiate transcription (Teng et al., 2005; Meng et al., 2020). CINV1 then hydrolyzes sucrose to generate glucose, the signaling cue. Glucose then negatively controls the stability of EIN3 *via* the glucose sensor HEXOKINASE1 (HXK1), which releases the suppression on the expression of *PAP1* and *TT8* from the YDA-EIN3 module (Meng et al., 2018, 2020). However, sugar-induced anthocyanin production by the HXK1-YDA/EIN3-MYB signaling cascades has been reported only in *A. thaliana* to date, whether it exists in other plants remains to be tested.

### Ethylene signaling pathway

In *Arabidopsis*, ethylene suppresses anthocyanin accumulation, which is substantiated by the higher levels of anthocyanins in ethylene-signaling mutants such as *etr1, etr2, ers1, ers2, ein2, ein3*, and *eil1* (Jeong et al., 2010). CONSTITUTIVE ETHYLENE RESPONSE1 (CTR1) acts as a negative regulator in ethylene signaling by directly phosphorylating EIN2, while ethylene inactivates CTR1 to trigger EIN2 to activate transcription of *EIN3* and *EIL1*, which subsequently turn on the expression of ethylene-responsive genes (Wang J. et al., 2015). Consistent with the signaling cascade, total anthocyanins were higher in *ein2* and *ein3/eil1* mutants but lower in *ctr1* mutants compared to wild-type levels (Jeong et al., 2010; Lei et al., 2011). Ethylene inhibits transcription of positive regulators including *TT8* and *PAP1/MYB75* through the activation of *EIN3* and promotes the expression of *MYBL2*, which encodes a negative regulator that forms an inactive transcriptional complex with TTG1 and TT8 (Meng et al., 2018). Therefore, this ethylene-mediated signaling cascade comprises CTR1-EIN3/EIL1-TT8/PAP1 to suppress anthocyanin accumulation in *Arabidopsis*.

In contrast to its action in *Arabidopsis*, ethylene promotes anthocyanin accumulation in apple fruits. In apple, anthocyanin levels are enhanced as ethephon concentration increases and repressed by ethylene inhibitor 1-methylcyclopropene (1-MCP; An et al., 2018). In the ethylene biosynthetic pathway, ACC synthase (ACS) and ACC oxidase (ACO) are critical enzymes for generating ethylene from *S*-adenosyl methionine. Ethylene production and anthocyanin levels are lower in MdACO1-silenced fruit peels compared with the wild type and higher in MdACO1-overexpressing fruit peels, in which MdACO1 generates ethylene to activate MdEIL1 expression, a homolog of EIN3 that promotes *MdMYB1* expression (Yu J. et al., 2022). Moreover, MdMYB1 and MdMYB10 each activate *MdACS* and *MdACO* expression by upregulating ethylene response factor (ERF) genes such as *MdERF106* and *MdERF3*, thereby promoting ethylene biosynthesis (An et al., 2018; Espley et al., 2019). Another component of the MBW complex, MdbHLH3, also promotes expression of the ethylene biosynthetic genes *MdACO1*, *MdACS1* and *MdACS5* by directly binding to their promoters, which then provides positive feedback to regulate ethylene biosynthesis and anthocyanin accumulation in apple fruit (Hu et al., 2019).

### JA signaling pathway

Jasmonates (JAs) induce anthocyanin accumulation in *Arabidopsis* (Shan et al., 2009), apple (An et al., 2015), pear (Ni et al., 2020) and tomato (Chen et al., 2022), and the underlying molecular mechanism has been widely investigated. In *Arabidopsis*, JASMONATE ZIM DOMAIN (JAZ) members negatively regulate the JA response by interacting with the protein MYC2, which is a positive regulator of JA signaling. JA stimulates the interaction of COI1 with JAZ proteins and then mediates the degradation of JAZs to release MYC2, which is essential for anthocyanin accumulation (Sasaki-Sekimoto et al., 2013; An et al., 2016). JASMONATE ASSOCIATED MYC2-LIKE1 (JAM1), JAM2, and JAM3 also negatively regulate JA-mediated anthocyanin accumulation by repressing *AtPAP1* expression, probably by competitively binding to the G-box, targeted by MYC2, in the *AtPAP1* promoter (Nakata et al., 2013; Sasaki-Sekimoto et al., 2013). Besides repressing MYC2 activity, JAZ proteins have been reported to inhibit anthocyanin biosynthesis by forming an inactive complex with the MBW components MYBs and bHLHs (Qi et al., 2011; An et al., 2015). Moreover, JAZs have recently been found to form a transcriptional repressor complex with an EAR motif-containing adaptor protein and co-repressor TOPLESS-RELATED 2 (TPR2), which reduces H3K9ac levels on the promoters of ABGs targeted by AtPAP1 (Li et al., 2020). Thus, the JAZ-MYC2 module plays critical roles in JA-mediated anthocyanin biosynthesis by regulating R2R3-MYB activators at the transcriptional and protein level (Figure 5). In plant disease resistance and apical hook formation of hypocotyl, JAZs and MYC2 act as integrators of ethylene and JA signaling by interacting with EIN3 and repressing transcriptional activity of EIN3, but the effect of the interaction on anthocyanin biosynthesis is unclear (Song et al., 2014). Because JA promotes anthocyanin production in *A. thaliana*, MYC2 might activate *AtPAP1* expression in part by repressing EIN3 activity. However, in mature pears, JA promotes ethylene production and induces anthocyanin accumulation in the absence of ethylene, indicating the dominant role of ethylene in anthocyanin biosynthesis (Ni et al., 2020). In addition, the JAZ-interacting DELLA proteins, negative regulators in GA signaling, interact with MYBL2 to form a DELLA-JAZ-MYBL2 complex and release an active MBW complex for anthocyanin biosynthesis in response to abiotic stresses (Xie et al., 2016). Collectively, an integrated network of JA and other hormones relay on JAZ-MYC2 modules to regulate anthocyanin accumulation in plants.

## Future perspectives

The R2R3-MYB is the master transcription regulator of the anthocyanin biosynthetic pathway, which is involved in intracellular signal transduction pathways to many types of extracellular environments. In this review, we outlined the upstream regulators of anthocyanin-related R2R3-MYB at multiple levels. In most cases, their regulation at the transcriptional or protein level has been demonstrated, but the exact mechanisms underlying their role in plant responses to various stimuli often have not been fully elucidated. Future research should focus on elucidating signaling crosstalk mechanisms that may fine tune the regulation of R2R3-MYB in different hormonal and stress conditions. For instance, COP1/PIFs-HY5/BBXs-MYBs signaling cascades are involved in transcriptional regulation of anthocyanin-related MYB activators from light and temperature cues (Figure 4), and the mechanism has been shown to be conserved in other plants. Several omics technologies present opportunities to identify new upstream regulators of R2R3-MYB activators. At the transcriptional level, candidate genes can be screened by co-expression and co-response analyses with R2R3-MYB using transcriptome sequencing (RNA-seq) under different conditions. At the protein level, protein immunoprecipitation and MS-based quantitative proteomic approaches will help identify new MIPs. Posttranslational modifications of R2R3-MYBs can also be characterized using tandem mass spectrometry and ion-electron reactions. Understanding the various regulatory mechanisms of R2R3-MYB will facilitate the design of strategies for molecular breeding programs using gene editing to develop novel crops, fruits, and vegetables with enhanced nutrition and health benefits.

## Author contributions

JY and YC drafted the manuscript and made tables and figures. JY and YW designed the review. YL, HS, and ZX provided comments and helped to write the final manuscript. All authors contributed to the article and approved the submitted version.

## Funding

The authors are grateful for the financial support provided by the Fundamental Research Funds for the Central Universities (2572022BA05), Natural Science Foundation of Heilongjiang Province of China (YQ2021C003), and Science and technology commissioners serve rural revitalization (2021NK4152).

## Conflict of interest

The authors declare that the research was conducted in the absence of any commercial or financial relationships that could be construed as a potential conflict of interest.

## Publisher’s note

All claims expressed in this article are solely those of the authors and do not necessarily represent those of their affiliated organizations, or those of the publisher, the editors and the reviewers. Any product that may be evaluated in this article, or claim that may be made by its manufacturer, is not guaranteed or endorsed by the publisher.
